# Evaluating lay first responder (LFR) first aid kit supplies usage and appropriateness in Western Kenya

**DOI:** 10.11604/pamj.2024.48.169.44049

**Published:** 2024-08-12

**Authors:** Ashwin Jitendra Kulkarni, Anagha Balaji Thiagarajan, Simon Ochieng Ogana, Dinnah Akosa Okwiri, John Arudo, Nathanael Smith, Zachary Eisner, Peter Delaney

**Affiliations:** 1 University of Michigan Medical School, Ann Arbor, Michigan, United States of America,; 2 Lay First Responders International, Los Angeles, California, United States of America,; 3 Michigan Center for Global Surgery, Ann Arbor, Michigan, United States of America,; 4 Department of Paramedical Sciences, Masinde Muliro University of Science and Technology, Kakamega, Kenya,; 5 Masinde Muliro University of Science and Technology, School of Nursing, Kakamega, Kenya,; 6 Department of Emergency Medicine, Washington University in St. Louis, St. Louis, Missouri, United States of America,; 7 Department of Orthopaedic Surgery, Cleveland Clinic, Cleveland, Ohio, United States of America

**Keywords:** Emergency medical services, first aid, first responders, Kenya, prehospital care

## Abstract

**Introduction:**

low- and middle-income countries (LMICs) disproportionately bear 90% of global mortality from trauma, yet robust emergency medical services (EMS) are often lacking to address the prehospital injury burden. Training lay-first responders (LFRs) is the first step toward formal (EMS) development in (LMICs). However, a gap remains as LFR first aid kit supply usage, appropriateness, and decay rates have yet to be studied but remain critical information for building sustainable LFR programs.

**Methods:**

we trained and equipped 101 LFRs in Kakamega County, Kenya in December 2023. During 3-month follow-up post-training, LFRs were surveyed with a 24-question multiple choice and free-response cross-sectional survey. Survey items included LFR demographics, patient encounters, first aid kit supplies usage, supply appropriateness, and local capacity for re-supply. Demographic data, usage statistics, appropriateness of current and potential kit additions, and local manufacturing capacity were collected and analyzed. Likert scales were utilized for categories consisting of “recommendation”, “potential recommendation”, and “not recommended” based on 100% - 75.0%, 74.9% - 60%, and 59.9% - 0% agreement, thresholds used in prior Delphi studies and meta-analyses. The survey design followed the Checklist for Reporting of Survey Studies (CROSS) guidelines to ensure quality standards.

**Results:**

of 101 total LFRs, 82 participated (82/101= 81.2% response rate). Participating LFRs were 80.5% men, and 65.9% had transportation-related occupations. LFRs reported 394 assisted incidents over three months (median= 4.0, IQR: 3.0, 5,0). Gloves, gauze/bandages, and towels were the most used supplies employed in 88.9%, 61.3%, and 34.7% of incidents, respectively. For current first aid kit item appropriateness, LFRs reached a consensus agreement on gloves (92.7%), gauze/bandages (91.5%), and towels (79.3%). For potential first aid kit additions, LFRs recommended alcohol wipes/hand sanitizer (89.0%) and tape (77.2%) but did not recommend water bottles or traffic cones. Lay-first responders (LFRs) agreed (90.2%) on the importance of local supply production and desired a streamlined resupply protocol.

**Conclusion:**

a survey on first aid kit supplies usage and appropriateness from Western Kenya demonstrated materials for body substance isolation, wound care, and hemorrhage management are critical to supply. Organized protocols for local materials resupply are essential to ensure program sustainability and continuity.

## Introduction

The global injury burden comprises six million deaths annually, representing 10% of global mortality, 90% of which is borne by low- and middle-income countries (LMICs) [1]. Road traffic injuries (RTIs) are the single largest contributor to global injury burden [2] and are also responsible for generating the highest number of injury-related disability-adjusted life years (DALYs) with LMICs comprising 24 of the 25 most affected countries [3,4]. Sub-Saharan Africa is projected to outpace the rest of the world in the growth of RTI-related mortality by 2030, with RTI´s expected to become the fifth-leading cause of death worldwide by the decade's end [5,6]. To address the disproportionate burden of prehospital injury faced by LMICs, the World Health Organization (WHO) suggests training lay first responders (LFRs) as the first step toward formal emergency medical services (EMS) development [7,8]. Despite the 2019 and 2023 World Health Assembly declarations that prehospital emergency care should be a global priority and that emergency, critical, and operative care services are critical for a comprehensive national primary healthcare approach to effectively address emergencies, little progress has been made by governments to build formal EMS capacity [9-11].

In Kenya, East Africa´s largest economy by gross domestic product (GDP), RTIs account for 27% of injury-related deaths [5]. Epidemiological analysis from a Kenyan tertiary care center suggests trauma patient characteristics are similar to other sub-Saharan LMICs, with a majority of patients being low-income, while the average interval between injury and hospital arrival is 70 minutes. This suggests there may be strategies that could be employed to prevent mortality [12]. Just 7% of Kenyan trauma patients receive prehospital care due to a lack of an integrated EMS system, public-access toll-free ambulance, and 24-hour emergency departments in level 4, 5, or 6 hospitals [13-15].

Though curricular development, dispatch, and subsequent patient impact of LFR programs have been extensively studied, supplies and equipment usage informing supply chain decisions are critical to inform further sustainable LFR program development. Prior LFR programs have utilized low-cost items to assist with basic life support (BLS) training themes such as scene safety, hemorrhage control, and fracture splinting with proven knowledge retention and clinical outcomes benefits [16-22]. Nevertheless, there is significant variation in first aid kit items across studies and LFR attitudes towards first aid kit supplies have not been studied. In the current study, we aim to evaluate LFR first aid kit supplies usage and appropriateness in Western Kenya by surveying LFR patient encounters, first aid kit supply usage, supply appropriateness, and local capacity for re-supply to inform future EMS supply chain development across LMICs.

## Methods

**Study design and bias control:** our cross-sectional cohort study was performed in March 2024 during a 3-month follow-up interval for an LFR program launched in Kakamega, Kenya in December 2023. The 3-month interval was selected to ensure minimal selection bias and informed by prior follow-up intervals between 3 to 6 months post-training [19-22]. The survey instrument (Table 1) was developed by the multi-national authorship team and piloted among a group of 12 LFR trainers from the same four sub-counties as trained LFRs (age range: 22-44 years old, 33% female) to ensure local appropriateness and comprehension. Feedback was implemented to improve survey literacy and add additional survey items they deemed useful. Our study followed the standardized Checklist for Reporting of Survey Studies (CROSS) guidelines to report findings and limit survey bias [23]. This study follows a preregistered Strengthening the Reporting of Observational Studies in Epidemiology (STROBE) reporting guidelines [24], minimizing reporting bias. The Institutional Review Board at the University of Michigan deemed our study to be exempt as our study was conducted during follow-up assessments for the Deploying Emergency Bystander Internet Training (DEBIT) Trial for First Responder Education.

**Table 1:** participant survey: evaluating Lay First Responder first aid kit supplies usage and appropriateness in Western Kenya

Question	Answer choices
1. What is your age range? (in years)	a. 21-30 b. 31-40 c. 41-50 d. 51-60 e. 60+
2. What is your gender?	a). Male b). female c). other
3. What is your occupation?	Free response
4. (If applicable) how many years of experience do you have as a transportation provider?	Free response
5. How many road traffic incidents have you witnessed in the past year?	Free response
6. How many people have you assisted in an emergency since training?	Free response
7. How many times have you used gloves to treat a patient?	Free response
8. Gloves are an important part of the LFR training kit.	a). Strongly agree b). agree c). neutral d). disagree e). strongly disagree
9. How many times have you used a towel to treat a patient?	Free response
10. Towels are an important part of the LFR training kit.	a). Strongly agree b). agree c). neutral d). disagree e). strongly disagree
11. How many times have you used a wooden board and fabric tie to treat a patient?	Free response
12. Wooden boards and fabric ties are an important part of the LFR training kit.	a). Strongly agree b). agree c). neutral d). disagree e). strongly disagree
13. How many times have you used gauze/bandages to treat a patient?	Free response
14. Gauze/bandages are an important part of the LFR training kit.	a). Strongly agree b). agree c). neutral d). disagree e). strongly disagree
15. How many times have you used a pen/tourniquet to treat a patient?	Free response
16. Pen/Tourniquets are an important part of the LFR training kit.	a). Strongly agree b). agree c). neutral d). disagree e). strongly disagree
17. Two bottles of water would be helpful additions to the LFR training kit.	a). Strongly agree b). agree c). neutral d). disagree e). strongly disagree
18. Alcohol wipes/hand sanitizer would be helpful additions to the LFR training kit.	a). Strongly agree b). agree c). neutral d). disagree e). strongly disagree
19. Tape would be a helpful addition to the LFR training kit.	a). Strongly agree b). agree c). neutral d). disagree e). strongly disagree
20. Printed out instructions detailing recovery position and triage would be helpful additions to the LFR training kit.	a). Strongly agree b). agree c). neutral d). disagree e). strongly disagree
21. Traffic cones would be a helpful addition to the LFR training kit.	a). Strongly agree b). agree c). neutral d). disagree e). strongly disagree
22. What are some items that you think would be helpful additions to the LFR training kit?	Free response
23. Producing kit supplies locally in Kakamega County is important.	a). Strongly agree b). agree c). neutral d). disagree e). strongly disagree
24. What are some LFR kit items that could be produced locally?	Free response

LFR: Lay First Responder

The survey contained 24 questions: 6 questions assessed LFR demographics, 7 questions were free response questions, and 11 questions utilized a Likert scale (strongly agree, agree, neutral, disagree, strongly disagree) to assess first aid kit supplies usage and appropriateness. Likert scale questions were assigned the following categories based on percent agreement: recommendation (75% - 100% answered strongly agree/agree), potential recommendation: (60% - 74.9% answered strongly agree/agree), and not recommended (0% to 59.9% answered strongly agree/agree). These thresholds have been well established across various medical fields, including neurology, hematology, and gastroenterology as well as a systematic review of 98 studies designed to analyze consensus [25-28].

**Setting and participants:** an initial needs assessment and appropriateness study was performed in 2023, suggesting LFR training might improve prehospital care in Kakamega, Kenya [29]. An LFR program was then established by Masinde Muliro University Department of Paramedical Sciences (MMUST) in partnership with LFR International. Training was administered in four of twelve sub-counties within Kakamega: Khwisero (n= 19 LFR participants), Lurambi (n= 27), Mumias East (n= 22), and Shinyalu (n= 14) in December 2023 using an LFR curriculum iteratively developed by LFR International during previous projects across Uganda, Chad, South Africa and Sierra Leone [16-22]. Based on the local needs assessment, we leveraged the pre-existing infrastructure of transportation providers through trained motorcycle taxi drivers given their wide geographic coverage secondary to self-dispersion in search of customers, profession-driven proximity to RTIs, and potential scalability of the LFR program post-implementation [17,18]. Each LFR training session consisted of 25 participants and was led by five primary TOT instructors. The course consisted of five modules: (1) scene management, (2) airway and breathing, (3) bleeding control, (4) fracture management, and (5) victim transport modeled off of prior LFR training programs [16-22].

**Study size:** in March 2024, we administered our survey to 82 LFRs in Kakamega, Kenya initially trained in the December 2023 pilot. The inclusion criteria for the study included trained LFRs who were initially chosen for training via a lottery system.

### Study variables and data measurement

**Lay First Responder (LFR) demographics:** six demographic questions were included spanning LFR age range, gender, occupation, and years of experience in their stated occupation. Participant-identifying information was not collected to ensure LFR anonymity.

**Incident reporting:** to better understand incident frequency, we asked participants about the number of RTIs they encountered in the past three months. We then asked LFRs the number of RTIs where they assisted bystanders.

**First aid kit supplies usage and appropriateness:** we then proceeded to include five item usage questions. These free-response questions asked participants how frequently they used the referenced kit item over the past three months to determine decay. The first question fell under the category “scene safety” to assess glove usage. The second question fell under the category “victim transport” to assess towel usage. The third question fell under the category “fracture splinting” to assess wooden splinting board usage. The fourth and fifth questions fell under the category “hemorrhage control” to assess gauze/bandage usage and pen/tourniquet usage.

**First aid kit supplies appropriateness:** our survey included five sentiment questions assessing kit item appropriateness following the corresponding kit usage question, asking LFRs if they agreed that the specific item is an important part of the kit based on their experience as LFRs. These questions utilized Likert items (strongly agree, agree, neutral, disagree, strongly disagree).

**New potential item sentiment questions:** our survey then included five sentiment questions relating to new potential kit items to determine a need for additional supplies not previously included in LFR kits. These questions also utilized Likert scales. New potential first aid kit additions included items that had appeared in previous BLS and prehospital trauma life support (PHTLS) curricula including two water bottles of water, alcohol wipes/hand sanitizer, tape, printed-out quick reference instructional sheets instructions (detailing recovery positions and triage instructions), and traffic cones which were derived from prior basic life support (BLS) and prehospital trauma life support (PHTLS) curricula [30-32]. A free-response question was also included for respondents to note and describe potential first aid kit additional items they felt would be useful.

**Local resource production questions:** to assess capacity for cost savings and local economic growth, we asked surveyed respondents a Likert scale question on the importance of manufacturing items locally in Kakamega, Kenya. We also included a free-response question for respondents to write what specific materials could be produced locally.

**Statistical methods:** survey data was documented and collected independently by two researchers simultaneously in March 2024 in Kakamega, Kenya to minimize error. Descriptive analysis was used for all non-Likert scale questions primarily with the categories of demographics and item usage categories. Given the non-parametric data, median and interquartile range were used as measures of central tendency and variance, respectively, for continuous variables. For all questions including Likert scales, responses were categorized by the number of respondents who indicated strongly agree/agree (falling in the “recommendation” category) for the necessity/importance of that item with consensus previously defined in the “study design” section. Considering the frequency of similar feedback, free response questions were analyzed using categorical analysis, an effective means of categorizing qualitative data with similar responses by allowing for the numerical frequency to represent the frequency of a certain response [33,34].

## Results

Of the total 101 trained LFRs, 82 (response rate: 81.2%) participated in the survey.

**Participants:** median participant age range was 36 - 45 years. In total, 80.5% (n= 61) were male and a majority (65.9%) worked as motorcycle taxi drivers (n= 51) or car taxi drivers (n= 3), with a median of 5 years of experience (IQR: 0.5, 8.8). Other occupations included public health (n= 19), farming (n= 3), and manufacturing (n= 1) (Table 2).

**Table 2:** participant demographics

Demographic category	N	Percent (%)
**Gender**		
Male	66	80.5
Female	16	19.5
Other	0	0
**Age (years)**		
18-29	20	24.3
30-39	21	25.6
40-49	20	29.2
50-59	18	26.8
≥60	3	3.7
**Sub county**		
Kwisero	16	19.5
Lurambi	26	31.7
Mumias East	21	25.6
Shinyalu	19	23.2
**Occupation**		
Boda boda driver	51	62.2
Public health worker	19	23.2
Taxi driver	3	3.7
Farmer	8	9.8
Manufacturing	1	1.2
**Years of transportation experience**		
0	21	25.6
1-5	30	36.6
6-10	17	20.7
11-19	12	14.6
≥20	2	2.4

### Main results

**Incident reporting:** lay first responders (LFR) witnessed a median of 5.0 (IQR: 3, 8.8) RTIs over the 3-month interval post-training, while assisting a median of 4.0 (IQR: 3.0, 5.0) bystanders. A total of 394 incidents were attended to by the study participants. On average, each LFR assisted 1.6 incidents per month and responded to an average of 77% of RTIs they witnessed. The top 7% of LFRs responded to at least 10 incidents over the three months.

**First aid kit supplies usage and appropriateness:** first aid kit usage varied widely by item. Over the three-month interval post-training, the median glove usage per respondent was 3.5 (IQR: 2.0, 5.0) times, gauze/bandages 2.0 (IQR: 1.0, 3.8) times, towels one (IQR: 1.0, 2.0) time, splinting board/fabric ties one (IQR: 0.0, 2.0) time, and pen/tourniquets one (IQR: 0.0, 2.0) time. By frequency, gloves were used in 88.9% of encounters, gauze/bandages in 61.3%, towels in 34.7%, and both splinting board/fabric ties and pen/tourniquets in 30.8% of encounters.

**Figure 1:**
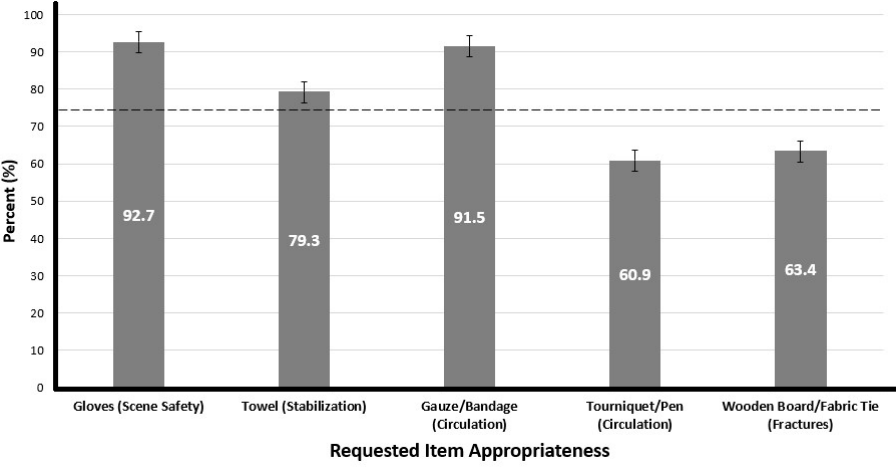
appropriateness of current kit items

Respondents were mixed in their perception of kit materials. In total, 92.7% of respondents agreed gloves, 91.5% agreed gauze/bandages, and 79.3% agreed towels were important additions to the LFR kit. These three items reached a consensus agreement and fell within the recommended category. Splinting board/fabric ties and pen/tourniquets did not reach consensus with 63.4% and 60.9% agreement, respectively, and fell within the potential recommendation category. No materials were listed in the not recommended category (Figure 1).

**Figure 2:**
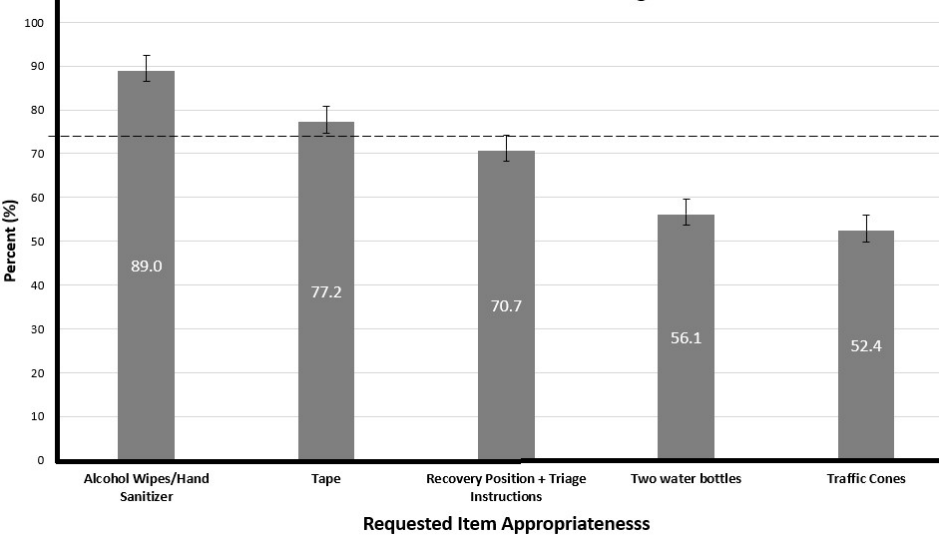
appropriateness of potential kit items

**New item sentiment questions:** lay first responders (LFR) reached a consensus on two potential first aid kit additions. The most popular item was alcohol wipes/hand sanitizer with 89.0% of respondents agreeing on its inclusion while 77.2% agreed tape should be included, qualifying these items for recommendation. Meanwhile, 70.7% of respondents agreed instructions detailing recovery position/triage should be included, placing this item in the potential recommendation category. Two bottles of water and traffic cones both fell within the not recommended category with 56.1% and 52.4% agreement, respectively. Our free response question indicated two frequently indicated additional items: alcohol/iodine (n= 23, 34.8%) and scissors (n= 17, 25.8%). Additionally, 37% (n= 24) of responses requested more frequent restocking of supplies, particularly in regard to gloves and gauze (Figure 2).

**Local resource production:** respondents reached a consensus, with a majority (89.0%, n= 73) agreeing local production of kit materials was important. On a free-response question specifying which items could be made in Kenya, the three most frequent answers were gauze/cloth/tourniquets (65%, n= 31), gloves (28%, n= 18), and kit bags (13%, n= 8). These results suggest local manufacturing should be considered for LFR training programs to reduce costs.

## Discussion

Our study provides the first analysis of LFR first aid kits in LMICs while adding detailed incident reporting data to existing literature. The data obtained includes nearly 400 incidents in three months following LFR training. Three conclusions are of particular importance. First, LFRs place an emphasis on hemorrhage management (HM) and wound control materials. Second, LFRs report less utility for high-level HM and fracture splinting materials, though they still show importance. Third, LFRs place emphasis on local kit manufacturing and cite a need for consistent and organized resupplies, especially among super-responders.

Our survey points to an increased importance on low-level HM and wound care. This is evidenced by LFR consensus on usage/sentiment on gloves and gauze/bandages, consensus addition on alcohol wipes, and further requests for fabric, scissors, and gauze resupply. This is also in line with prior studies where LFRs have previously reported HM as their most frequently utilized skill, employed in over 60% of patient encounters [11,16]. Gloves and gauze/bandages are the most used supplies, employed in 90% and 63% of encounters, respectively, thus establishing themselves as essential materials with consensus agreement among LFRs. Trauma scissors and tape should be considered for inclusion as prior studies have supported their use as a cost-effective means for wound access and skin closure [35,36]. Additionally, many LFRs personally added this item to their kit, demonstrating both its essential nature and the responder's willingness to provide some amount of financial contribution to kit maintenance. Most importantly, our study found hand hygiene materials are currently inaccessible to responders and that LFRs reached a consensus agreement that hygiene materials should thus be provided in first aid kits. While these materials are known to decrease address infection vector rates and increase overall outcomes in high-income country hospitals, healthcare-associated infection (HAI) rates are three times higher in LMICs largely due to a lack of access [37,38]. In a prospective study in another LMIC, Brazil, researchers found a lack of hand hygiene in 93% of HAIs while the Occupational Safety and Health Administration (OSHA) in the US estimates that 1.5 million of the 6 million EMS workers are at risk for bloodborne pathogens [38-40]. The data remains clear: LFRs in LMICs frequently encounter wounds as well as low-level HM incidents in line with evidence showing that 93% of prehospital hemorrhage-related deaths occur in LMICs [41]. Recommended items for these injuries include gloves, gauze, fabric, tape, scissors, and alcohol wipes/hand sanitizer.

In contrast to the prevalence of low-level HM and wound control, LFRs placed decreased emphasis on materials for high-level HM and fracture splinting. Tourniquets/pens were applied only half as frequently, at 31% usage, as compared to basic HM materials such as gauze/bandages at 63% usage. This resulted in LFRs not reaching a consensus agreement on the need for tourniquet inclusion in LFR first aid kits. This points to a novel study finding; while previous LFR studies have demonstrated HM as the most frequently used skill, the distinction between low-level HM and high-level HM has yet to be investigated [11,17-21]. Nevertheless, tourniquets should be included in LFR kits as they are used in a third of incidents, have reduced mortality, and even boast an 81% success rate in bleeding cessation when applied by lay people [11,17-21,42,43]. Similar to tourniquets, LFRs also did not reach a consensus on the inclusion of wooden fracture splints which were used in 31% of incidents. Prior research studies have found utility for extremity fracture splinting to reduce pain and blood loss, but only if doing so does not delay patient transport [44]. This contrasts with C-spine immobilization with the LFR towel which has proven non-inferiority to a cervical collar, consensus efficacy agreement among LFRs, and previous literature supporting its importance regardless of its impact on transportation times [45,46]. Once again, the inclusion of wooden splinting boards may be warranted if feasible due to frequent use and minimal costs. However, a debate is warranted as these conclusions may suggest that less LFR training time should be dedicated to high-level HM and fracture splinting with increased attention paid to low-level HM and wound control.

Third, LFRs place emphasis on local kit manufacturing and cite a need for consistent and organized resupplies, especially among the highest responding quartile of responders, reaching a consensus on the importance of local material manufacturing. Gauze/cloth, gloves, and first aid kit bags were suggested as items that should be considered for local production. This is a realistic prospect as Kenya´s USD$400 million textile industry employs 50,000 individuals and is a major contributor to female workforce participation [47]. LFRs also seek to increase capacity for sustainable LFR kit resupply, a concern voiced by 37% of respondents. LFR programs have previously ensured sustainability through a trainer-of-trainers (TOT) model, robust local partnerships, and combined international and domestic funding [16-22]. Resupply of kit items including gloves, fabric, and now alcohol wipes is a crucial next step as LFRs have reported depleting their initial supply of gloves and gauze running out after just three incidents. Literature on the frequency of kit resupply is lacking; however, a program that should seek to employ strong local partnerships, consistent follow-up time intervals, and frequent digital communication is an ideal way to ensure adequate sourcing of LFR supplies for LFRs. Resupply rates will vary significantly among LFRs, given that the top 7% of LFRs disproportionately responded to 28% of incidents, supporting a previously described “super-responder” phenomenon described previously [48]. As a result, LFRs will require various frequencies of resupply due to their differing incident reporting involvement. For this reason, communication between TOTs, LFRs, and local partners on the usage of supplies is of high importance to ensure that super-responders are able to obtain supplies more frequently than their peers. Further evidence of a super-responder phenomenon in Kenya is a critical finding in our study and allows us to advocate for as-needed rather than scheduled resupply.

**Limitations:** our study is limited by its retrospective, cross-sectional survey-based design. First aid kit supplies decay rates across a cohort of LFRs merit prospective study, and future investigations should incorporate “supplies used” into patient encounter incident reports. This will not only inform resupply but also provide further insight into prehospital care provided by LFRs. Another primary limitation is the sample size of survey respondents, particularly regarding numerical and geographical diversity. Conclusions may appear limited in a sample of 82 responders across four sub-counties in Western Kenya. However, we remain confident in our data for various reasons. First, LFRs have attended to nearly 400 incidents, reassuring our incident sample size. Second, out of the four total sub-counties, two (Shinyalu and Mumias East) are considered rural while two (Lurambi and Khwisero) are considered urban, thus providing important geographic variance to supply usage. Third, the demographic diversity, with a fifth of respondents being women and over a third working in industries other than transportation, brings further reassurance on the external validity of the study. A secondary limitation pertains to non-response bias relating to our two free response questions on potential first aid kit additions and the importance of local materials sourcing, which had 66 and 64 responses for additional items and local sourcing questions, respectively, compared to 82 responses for all multiple-choice questions. This could be of potential concern, though we remain assured by the fact that a total of 146 and 136 individual items were tallied since as many LFRs listed multiple items in their responses, suggesting saturation with 80% and 78% response rates. Additionally, response bias is a potential concern as we retrospectively assessed first responder incident reporting. To minimize this bias, we assessed LFRs just three months following initial training, a method used in prior LFR studies which may have also contributed to a high response rate [19-22]. Lastly, survey studies are known to be potential victims of response, social desirability, and conformity bias [49]. To minimize this risk, we followed the validated CROSS guidelines prior to survey design [23] and followed the STROBE guidelines [24] for manuscript preparation.

## Conclusion

An analysis of LFR kit materials from 394 total incidents in Western Kenya revealed an emphasis on HM, wound care, and a need for an organized protocol for local material resupply. No current kit items met the criteria for exclusion. Alcohol wipes and tape were two potentials first aid new kit items additions that met consensus for inclusion. Training estimates for future LFR interventions should include funds for resupplying gloves, gauze, and alcohol wipes. Local healthcare and manufacturing partners should play a key role in the distribution and maintenance of LFR first aid kit materials. Future studies should prospectively study first aid kit supplies decay rates by incorporating “supplies used” into patient encounter incident reports.

### What is known about this topic

- *Low- and middle-income countries (LMICs) bear the majority of the global injury burden with LFRs being a proven mechanism to build EMS capacity;*
- *Prior LFR training programs have demonstrated knowledge retention and incident reporting in developing low-cost and effective basic life support training;*
- *There is a lack of knowledge on the usage and effectiveness of current and potential items in LFR first aid kits*.

### What this study adds

- *A detailed breakdown of the usage and appropriateness of current first aid kit items based on LFRs who responded to nearly 400 incidents;*
- *Recommendations for potential new kit items, local production capacity, and budget for frequent kit resupply;*
- *Lay First Responders (LFRs) cite an increased importance on wound control and low-level hemorrhage management, with decreased emphasis on high-level hemorrhage management and fracture splinting*.
